# Epidemiological characteristics and trends of hand-foot-mouth disease in Shanghai, China from 2011 to 2021

**DOI:** 10.3389/fpubh.2023.1162209

**Published:** 2023-05-30

**Authors:** Jing Wang, Shan Zhang

**Affiliations:** Department of Immunization Program, Huangpu District Center for Disease Control and Prevention, Shanghai, China

**Keywords:** HFMD, epidemiology, EV71 vaccine, vaccination status, seasonal trend

## Abstract

Hand, foot and mouth disease (HFMD) is a kind of infectious disease caused by enterovirus infection. In this study we analysed the epidemiological characteristics and time trends of HFMD, vaccination status and vaccine protection effect assessment of EV71 vaccine from 2011 to 2021 in Huangpu District, Shanghai, China. HFMD cases showed a decreasing trend year by year from 2011 to 2021, from 122 cases reported in 2012 to 7 cases in 2020, and 12 cases in 2021. Etiological diagnosis was CV-A6 in 185 cases (29.8%), CV-A16 in 209 cases (33.7%), EV-A71 in 118 cases (19.0%) and other enteroviruses in 109 cases (17.6%). After the launch of EV71 vaccine, a total of 32,221 doses of EV71 vaccine were administered between 2016 and 2021. The case–control results showed that there was no evidence to support the effectiveness of EV71 vaccine, OR (95% CI) =0.52 (0.12 ~ 2.3), *p* = 0.37. The epidemic strains have changed. Surveillance and management of HFMD remain very important in the future and EV71 vaccine is considered to be included in National Immunization Program.

## Introduction

Hand, foot and mouth disease (HFMD) is a kind of infectious disease caused by enterovirus infection, which mainly manifests as ulcerative herpes on the oral mucosa and blister-like rash on the extremities, which is common in children (1, 2). HFMD was listed as a notifiable infectious disease by the Ministry of Health of China in 2008 after an outbreak of HFMD which caused 23 deaths (3). The main pathogenic serotypes of HFMD include Coxsackievirus (CV) 4–7, 9, 10, 16 in group A, 1–3, 5 in group B, Enterovirus A71 (EV-A71) and partial serotypes of Echovirus. The common pathogens of HFMD in China are EV-A71, CV-A16 and some other enteroviruses (4, 5). However, the proportion of CV-A6 infections has gradually increased in recent years in China and around the world (6–8).The median annual incidence of HFMD was 153.78 per 100,000 (ranging from 120.79 to 205.06) in mainland China from 2011 to 2018 (9). China accounted for 87% (9.8 million/11.3 million) of all hand, foot, and mouth disease (HFMD) cases reported to WHO during 2010–2014 (10). EV-A71 is responsible for most of the severe HFMD cases and had the highest disease burden (11). HFMD not only costs, but also causes physical inconvenience and psychological pain to patients and their families. The invisible burden of these negative effects cannot be ignored. In 2016, Enterovirus Type 71 vaccine (EV71 vaccine) was approved for marketing in China (4). Currently, there are three manufacturers of EV71 vaccine on the market, including Wuhan Institute of Biological Products Co., Ltd. (Wuhan), Sinovac Biotech Co., Ltd. (Sinovac) and Institute of Medical Biology of Chinese Academy of Medical Sciences (CAMS) (12). In this study we analyzed the epidemiological characteristics and time trends of HFMD, vaccination status and vaccine protection effect assessment of EV71 vaccine in Huangpu District, Shanghai, China from 2011 to 2021. Our results can be used to adjust the prevention and control measures of HFMD and vaccination strategies.

## Methods

### Data collection

#### Exclusion and inclusion criteria

All HFMD cases were reported in China Disease Prevention and Control Information System due to the Law on the Prevention and Treatment of Infectious Diseases in China from 2008.

- Those who meet the diagnostic criteria of HFMD should be included, according to the National Health Commission’s Diagnosis and Treatment Guidelines for Hand, Foot and Mouth Disease.
- The time of onset was 2011–2021.
- Cases of HFMD have been laboratory diagnosed.

1. The specific PCR test of enterovirus (CV-A16, EV-A71, etc.) was positive.
2. Enterovirus was isolated and identified as CV-A16, EV-A71 or other enterovirus that can cause HFMD.

- Exclude clinically diagnosed cases without laboratory diagnosis.

Case information included population classification, age of onset, sex, onset time, diagnosis time, laboratory diagnosis, and pathogen classification. In the case reporting information system, the population was divided into: scattered children, preschool children, students and adults. In China, children who have not reached the age of kindergarten are usually referred to as scattered children.

### Laboratory testing methods

The stool samples of HFMD cases were collected according to the “Technical Scheme for Collection and Testing of Hand, Foot and Mouth Disease Specimens,” and the etiological detection and typing were carried out by real-time PCR method. The etiological test results were divided into: EV-A71, CV-A16, CV-A6 and other enteroviruses.

### Vaccination record

EV71 vaccination in Huangpu District of Shanghai from 2011 to 2021 was collected to analyze the relationship between EV71 vaccination and HFMD incidence trend. For HFMD laboratory-confirmed cases, the EV71 vaccination records were obtained by querying the Shanghai Immunization Information System. The full course of EV71 vaccination requires 2 doses, spaced 1 month apart. In this study, the completion of 2 doses of vaccination within 42 days before the onset of the disease was considered as a history of EV71 vaccination. Cases received one dose and vaccinated within 42 days before the onset of HFMD were considered to have no vaccination history. For some cases, if the vaccination is not in Shanghai, the vaccination record cannot be acquired in the Shanghai Immunization Information System. Therefore, the vaccination history of those cases was unknown and recorded as missing value.

#### Statistic methods

The cases were summarized and analyzed according to the time of onset and type of diagnosis, and the characteristics and trends of HFMD during 2011–2021 were described. Chi-square test was used to compare the differences of HFMD cases among different factors. According to the laboratory diagnosis results, the case group was diagnosed with EV-A71, and the control group was diagnosed with other enteric pathogens. Whether there was a history of EV71 vaccination as a risk factor, a case–control study was conducted to analyze the protective effect of EV71 vaccine. All statistical analyses were performed using SPSS version 18.0, *p* < 0.05 or 95% confidence interval (95% CI) excluding 0 was considered statistically significant.

## Results

### Epidemiological characteristics

From 2011 to 2021, a total of 621 laboratory-confirmed cases of HFMD were reported in Huangpu District, Shanghai, and no severe and fatal cases were reported. The mean age at onset was 4.1 years, the median age at onset was 3.7 years, the youngest was 2 months old, and the oldest was 26 years old. There were 368 males (59.3%) and 253 females (40.7%), with a sex ratio of 1.45:1. 290 (46.7%) were preschool children, 257 (41.4%) were scattered children, 73 (11.8%) were students, and 1 (0.1%) was an adult. 415 people (66.8%) had not received EV71 vaccine, 72 people (11.6%) had received EV71 vaccine, and 134 people (21.6%) could not find the vaccination records.

### Seasonal trend

**Figure 1:**
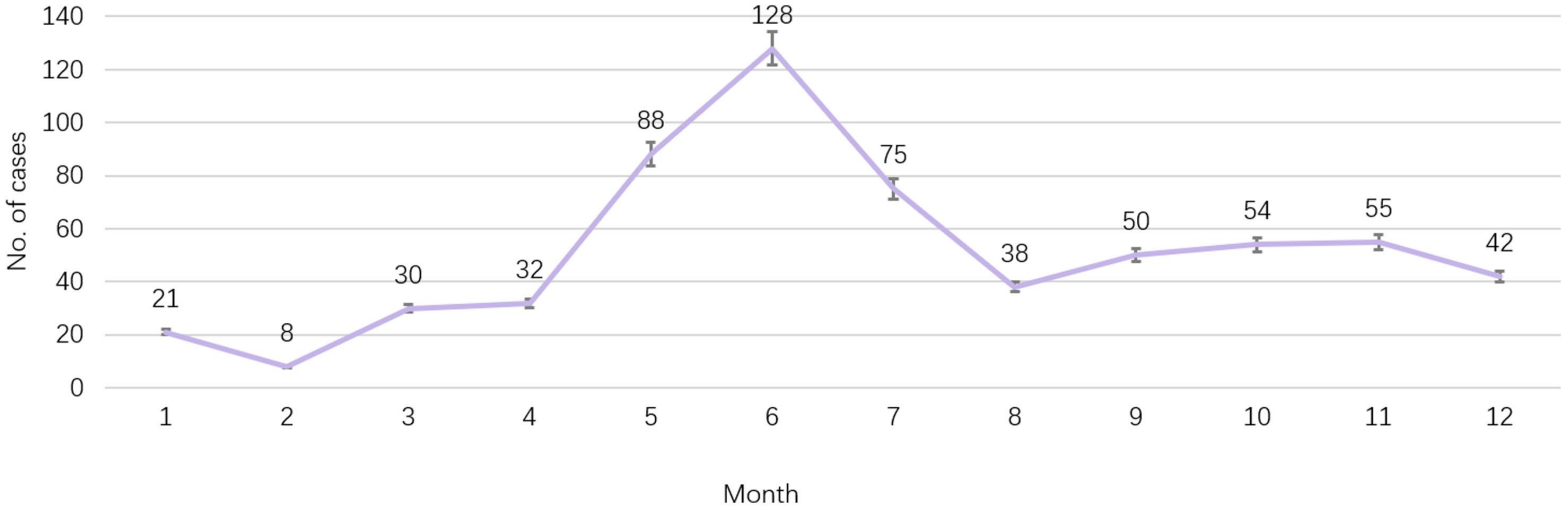
Seasonal trend of report cases of HFMD in Huangpu District, Shanghai, China from 2011 to 2021.

According to the reported onset year, HFMD cases showed a decreasing trend year by year from 2011 to 2021, from 122 cases reported in 2012 to 7 cases in 2020, and 12 cases in 2021. Among them, EV-A71 HFMD cases were reported most in 2011, with 37 cases, and then showed a downward trend. There was no EV-A71 HFMD case from 2018 to 2021. The month with the least reported cases was February (8 cases, accounting for 1.3%), the main peak of number of reported cases was from May to July, the second peak was from September to November. Most cases were reported in June (128 cases, 20.6%), May (88 cases, 14.2%) and July (75 cases, 12.1%) as shown in Figure 1.

### Etiological diagnosis

**Figure 2:**
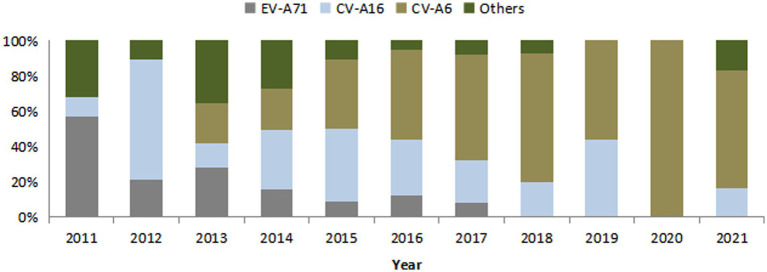
Composition of pathogenic diagnostic classification of HFMD cases in Huangpu District, Shanghai, China from 2011 to 2021.

Etiological diagnosis was CV-A6 in 185 cases (29.8%), CV-A16 in 209 cases (33.7%), EV-A71 in 118 cases (19.0%) and other enteroviruses in 109 cases (17.6%). The proportion of EV-A71 decreased year by year, while that of CV-A6 increased year by year. All cases reported in 2020 (7 cases) were diagnosed as CV-A6, and the specific composition ratio was shown in Figure 2.

The mean age of onset for each etiological diagnosis type was as follows: 4.4 years for CV-A6, 4.1 years for CV-A16, and 3.7 years for EV-A71 and other enteroviruses. The mean age of onset was statistically significant in the classification of etiological diagnosis, *F* = 3.02, *p* = 0.03. The influencing factors of gender, EV71 vaccination history and different population classification on the composition of etiological diagnosis types were analyzed, and the results were shown in Table 1. The influencing factor of EV71 vaccination history on the composition of etiological diagnosis types was statistically significant (*p* < 0.05).

**Table 1:** Composition and influencing factors of diagnostic classification of HFMD cases from 2011 to 2021.

	Etiological diagnostic classification								*χ*^2^	*p*
	CV-A6		CV-A16		EV-A71		Others			
	No. of cases	%	No. of cases	%	No. of cases	%	No. of cases	%		
Gender										
Male	103	55.7	122	58.4	74	62.7	69	63.3	2.37	0.50
Female	82	44.3	87	41.6	44	37.3	40	36.7		
EV71 vaccination history										
Yes	44	23.8	22	10.5	2	1.7	4	3.7	31.80	0.00
No	123	66.5	152	72.7	72	61.0	68	62.4		
Missing	18	9.7	11	5.3	111	94.1	113	103.7		
Population classification										
Adult	0	0.0	1	0.5	0	0.0	0	0.0	22.23	0.01
Scattered children	65	35.1	94	45.0	47	39.8	51	46.8		
Students	37	20.0	20	9.6	9	7.6	7	6.4		
Preschool children	83	44.9	94	45.0	62	52.5	51	46.8		
Total	185	100.0	209	100.0	118	100.0	109	100.0		

### Vaccination status

**Figure 3:**
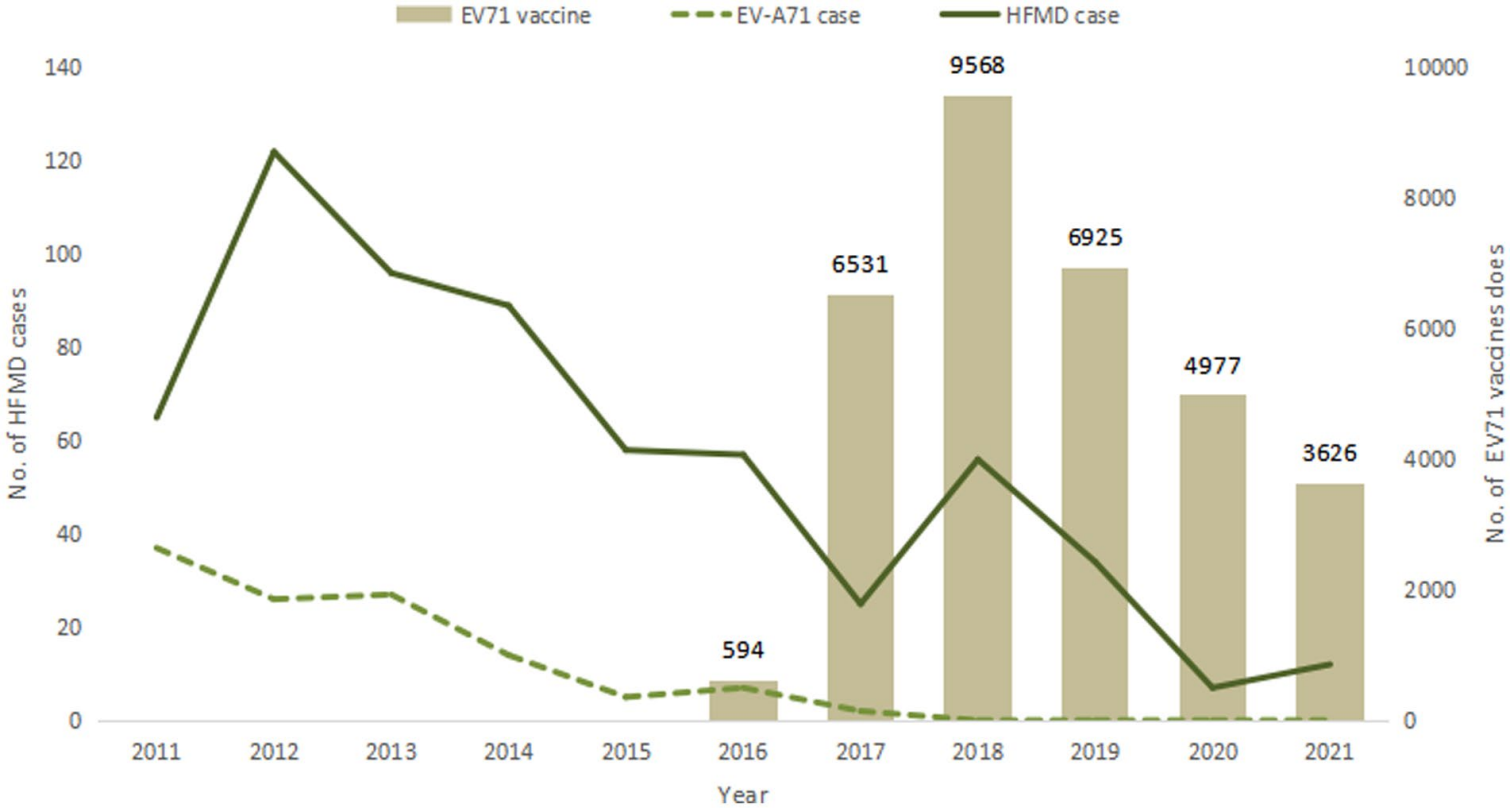
EV71 vaccination and HFMD case reports from 2011 to 2021.

After the launch of EV71 vaccine, a total of 32,221 doses of EV71 vaccine were administered between 2016 and 2021, with the largest amount of 9,568 doses in 2018. The vaccines were produced by three manufacturers, including 23,007 doses (71.4%) of CAMS vaccine, 6,120 doses (19.0%) of Wuhan vaccine, and 3,094 doses (9.6%) of Sinovac vaccine. The number of HFMD cases was compared with the amount of vaccination as shown in Figure 3.

### Effectiveness of EV71 vaccine

According to the etiological diagnosis classification, the confirmed EV-A71 cases were used as the case group, other cases were used as the control group, and the risk factor was effective EV71 vaccination history for case–control analysis. The proportion of vaccination history in the case group was 2.7%, and the proportion of vaccination history in the control group was 5.1%. The case–control results showed that there was no evidence to support the effectiveness of EV71 vaccine, OR (95% CI) was 0.52 (0.12 ~ 2.3), *p* = 0.37.

## Discussion

HFMD cases showed a decreasing trend year by year from 2011 to 2021.After the launch of EV71 vaccine. The epidemic strains have changed. The proportion of CV-A6, CV-A16 and other enterovirus types increased significantly. The case–control results showed that EV71 vaccination had no effect on the incidence. Surveillance and management of HFMD remain very important in the future and EV71 vaccine is considered to be included in National Immunization Program.

HFMD can occur throughout the year, with obvious seasonal characteristics, and the overall bimodal distribution. The results of this study are consistent with southern China, Hong Kong, and Taiwan (13–15). May to July is the main peak of HFMD incidence, and September–November is the secondary peak, probably because the distribution of enteroviruses is related to changes in natural factors such as seasons, climate, and humidity (13, 16). The incidence level decreased significantly in August, which may be related to the summer vacation and the reduction of children’s gathering in schools. In this study we found that the bimodal pattern of HFMD prevalence did not change after vaccination, suggesting that enterovirus infection is affected by climate, and vaccination of EV71 vaccine did not affect this pattern. From 2020 to 2021, there were only 7 and 12 HFMD cases, and no peak was observed, which was related to the implementation of COVID-19 prevention and control measures. Beginning in 2020, with the pandemic of COVID-19, gathering and movement have greatly reduced, and a series of personal protective measures have been implemented, such as wearing masks, strengthening hand hygiene, and maintaining social distance, classroom ventilation and environmental disinfection measures. These series of measures have raised public health awareness and improved healthy behaviors, reducing the occurrence of HFMD to a certain extent.

The male–female sex ratio of HFMD cases was 1.45:1, which may be because boys are active and more willing to participate in outdoor activities, which greatly increases the probability of exposure and infection, but there is no statistically significant difference in the pathogenic test results between males and females, indicating that the population is generally susceptible to HFMD. HFMD cases were mainly children in kindergartens and scattered children, accounting for 88.08% which was consistent with the results of other studies taken in China (27 months of age, 2008–2012) (13), Taiwan (under 4 years old, 1998–2005) (14), Hangzhou, China (preschool children, 2016–2018) (17) and Singapore (under 4 years old, 2001–2007) (18). This is due to the fact that young children have low immunity level, poor hygiene awareness and habits, and are more susceptible to enteroviruses (13, 19), resulting in the spread of HFMD. Infants under 6 months of age have fewer infections, most likely because of the protection of maternally transmitted antibodies (20, 21). And adults can acquire durable immunity through latent infection (13).

EV71 vaccine, as the first HFMD vaccine independently developed by China and launched in the world. It is currently the only vaccine that can effectively prevent HFMD, which made an important step in the prevention and treatment of HFMD. The EV71 vaccine is mainly suitable for children aged 6 months to 5 years old. The basic immunization program is two doses with a one-month interval in between. At present, EV17 vaccine is not included in the National Immunization Program (NIP), and the amount of vaccination doses are relatively small compared to other NIP vaccines. Many children even receive only one dose instead of the recommended two doses (19). The vaccination coverage of EV71 vaccine may increase if the vaccine was included in NIP and the vaccine costs covered by medical insurance in the future. This study showed that in 2011, EV-A71 virus was the main causative pathogen of HFMD. After the launch of EV71 vaccine in 2016, the HFMD etiological monitoring results showed that the epidemic strains have changed, and the proportion of EV-A71 type HFMD decreased significantly and no EV-A71 cases after 2018. Meanwhile, the proportion of CV-A6, CV-A16 and other enterovirus types increased significantly. Currently, the proportion of other enteroviruses such as CV-A6 in HFMD cases is increasing in China and worldwide (7, 22, 23). Thus, continuous monitoring of HFMD pathogen spectrum should be carried out, monitoring of CV-A6, CV-A16 and other enteroviruses should be strengthened and emphasized.

After school-age children are vaccinated against EV-A71, they can obtain personal immunity, thereby forming an immune barrier in the population and preventing the spread of EV-A71 virus in the population. Pre-market clinical trials of the vaccine have shown that vaccination of EV71 vaccine can prevent more than 90% of HFMD caused by EV-A71 virus, and the protection rate against severe HFMD can reach 100% (24–26). Although the results of case–control analysis showed no statistically significant difference, EV71 vaccine had no obvious protective effect on HFMD, but no EV-A71 type HFMD occurred since 2018, suggesting that there may be population protective effect of EV71 vaccine (6, 27). An immunogenicity analysis of EV71 vaccine in healthy children showed that the seropositive rate of was still 94.34% after 5 years of vaccination, indicating good persistence of the immunity generated by vaccination (28). Since EV71 vaccine does not have cross-protection effect against other viruses (24, 27, 29), children are still at risk of infected by other serotypes of HFMD after vaccination of EV71 vaccine, and this risk should not be ignored. The results of epidemiological studies on HFMD showed that CV-A6 and CV-A16 were two major non-EV-A71 enteroviruses causing HFMD, Xiao Dan Meng’s study in Hubei, China in 2016–2017 showed that CV-A6 accounted for 59.54% (30), Jiratchaya Puenpa’s study in Thailand in 2012 also showed that CV-A6 was the dominant pathogen accounted for 33.5% (31), and Thi NguyenHoa-Tran’s study showed that CV-A6 and CV-A16 were two major non-EV-A71 enteroviruses causing HFMD in Vietnam, 2008–2017 (32). At present, the pathogenic spectrum of HFMD-associated enteroviruses has changed, CV-A6, CV-A10 and other serotypes have gradually replaced EV-A71 and CV-A16 as the main pathogens. The EV71 vaccine cannot prevent HFMD caused by other serotypes. Faced with the changes of HFMD epidemic strains, the development of multivalent vaccines containing EV-A71 and other major serotypes has become a recognized means of preventing HFMD, controlling the spread of enteroviruses, and delaying the emergence of mutations and recombinant strains (33). Bivalent (CV-A6 + A10, EV-A71 + CV-A16) and trivalent (EV-A71 + CV-A16 + A6 and CV-A16 + A6 + A10) vaccine candidates are currently in clinical trials (34–36). In addition to traditional inactivated vaccines and live attenuated vaccines, there are also technical routes such as VLP vaccines, recombinant VP1 protein vaccines, and synthetic peptide vaccines (12).

Research on multivalent vaccines for HFMD will continue to rely on ongoing monitoring of enterovirus epidemiology and changes in pathogenic spectrum. Therefore, the surveillance and management of HFMD remain very important in the future. Currently, HFMD management in Shanghai mainly relies on pediatric outpatient clinics in large general hospitals for case reporting and sampling. Starting from 2022, pediatric outpatient clinics in community health service centers will open, which will improve the timeliness and scope of case detection and surveillance to a certain extent. At the same time, it is more important to evaluate the quality of surveillance work and training of staff in pediatric outpatient clinic.

## Limitations

The reported number of HFMD cases is only a part of the actual number of infections and cases in the population. It may be because the symptoms after infection are mild and they do not go to the hospital, and the EV-A71 type HFMD in the population has decreased significantly. As HFMD cases primarily occurred in children, the decreasing time trend reported by case counts might be associated with the decreasing trend of the total number of children in Shanghai, which might not reflect the actual temporal trend. Therefore, the evaluation of the protective effect of the vaccine requires further research.

## Data availability statement

The raw data supporting the conclusions of this article will be made available by the authors, without undue reservation.

## Ethics statement

This study was approved by the Ethics Review Board of Huangpu District Center for Disease Control and Prevention (No. 2022HPLL01). Anonymity was guaranteed. All methods were performed in accordance with the relevant guidelines and regulations. Consent to participate is not applicable.

## Author contributions

JW and SZ wrote the main manuscript text and JW prepared all figures and tables. All authors contributed to the article and approved the submitted version.

## Funding

This work was supported by Yangtze River Delta Regional Leading Talents Research Project on Immunization (CSJP032).

## Conflict of interest

The authors declare that the research was conducted in the absence of any commercial or financial relationships that could be construed as a potential conflict of interest.

## Publisher’s note

All claims expressed in this article are solely those of the authors and do not necessarily represent those of their affiliated organizations, or those of the publisher, the editors and the reviewers. Any product that may be evaluated in this article, or claim that may be made by its manufacturer, is not guaranteed or endorsed by the publisher.
